# IoT-enabled solutions for paediatric diabetes: examining consumer readiness and adoption factors

**DOI:** 10.3389/fdgth.2026.1747032

**Published:** 2026-07-08

**Authors:** Mohammad Al Nawayseh, Ghazi Al-Naymat, Dania Khraisat, Heba Al-Slehat

**Affiliations:** 1 Artificial Intelligence Research Center, College of Engineering and Information Technology, Ajman University, Ajman, United Arab Emirates; 2 Management Information Systems Department, Business School, University of Jordan, Amman, Jordan

**Keywords:** digital health, fsQCA, internet of things, mixed methods, paediatric diabetes, PLS-SEM, technology acceptance model

## Abstract

**Background:**

Paediatric diabetes management can be improved using emergent technological solutions, such as the Internet of Things (IoT). IoT systems can collate instant data from sensors, whereby service users and Healthcare Professionals (HCPs) can undertake blood glucose tracking in real time, reacting promptly when needed. However, adoption facilitators in practice are poorly understood.

**Research objective:**

To investigate healthcare adoption potential for paediatric diabetes care related to current IoT sensor technology.

**Research methods:**

Based on the Technology Acceptance Model as the theoretical framework, this study uses mixed methods. A quantitative survey of 90 parents, concerning IoT is analysed with Fuzzy-Set Qualitative Comparative Analysis (fsQCA) and Partial Least Squares Structural Equation Modelling (PLS-SEM) is combined with qualitative thematic analysis of semi-structured contextualising interviews with 10 paediatric HCPs.

**Results:**

PLS-SEM analysis revealed that perceived ease of use and usefulness positively affect user predispositions and willingness to adopt IoT solutions in paediatric diabetes care, reaffirmed by fsQCA outcomes affirming that high positive attitudes and usefulness support use albeit perceived ease of use is low. These insights are reaffirmed by interview outcomes, which underscore the importance of usability, integration with existing systems, and concern about data access and privacy.

**Implications:**

The outcomes indicate that technological system design (i.e., designers and health policy and managerial stakeholders) must prioritise clinical utility and impacts and not myopically focus on ease of use *per se*, providing necessary training and consulting users in order to effectively integrate technologies into existing systems and ward contexts. Behavioural and configurational analyses can be used to apprize sophisticated interactions and behaviours of adoption in health contexts.

**Conclusions:**

The outcomes of this research provide quantitative and qualitative support for HCPs' attitudes and the perceived usefulness of IoT technologies being key drivers of their adoption in diabetes management.

## 1 Introduction

Healthcare has always been at the forefront of technology adoption, including the recent paradigmatic breakthroughs of Machine Learning (ML) and the Internet of Things (IoT), which have expanded the scope for patient-centred service provision, including increasing access due to frequent or even real-time monitoring via remote sensors (1). Monitoring using contemporary digital technologies has revolutionised care options for chronic complaints, including cardiovascular diseases, hypertension, and diabetes, all of which can benefit from such tools and rapid health team responsiveness (2).

In the case of paediatric diabetes (ped-D), the stakes are particularly high. Diabetes impacts on paediatric and adolescent patients can have more serious immediate and lifelong impacts on various health and life outcomes. The prevalence of type 1 among paediatric is increasing by about 3% annually (41), and diagnosing the condition as early as possible is essential to optimise patients' prospects, along with appropriate glucose monitoring and lifestyle management (for the whole—hopefully long and healthy—lifespan) (3). IoT-based technologies hold great potential for ped-D management by facilitating real-time monitoring, proactive interventions, and better coordination between caregivers and HCPs (4, 5). However, despite significant technological advancements, there is a crucial gap in understanding how IoT-enabled systems can be effectively implemented, adopted, and scaled in real-world contexts, specifically for managing ped-D. Much of the existing literature concentrates on technical development rather than user adoption or contextual suitability. Furthermore, empirical studies examining the perceptions and readiness of end-users, particularly parents and HCPs, who are key stakeholders in the care of diabetic children, are notably lacking.

This paper seeks to address this gap by proposing and evaluating a comprehensive framework for intelligent health monitoring in ped-D care, integrating technological architecture with behavioural analysis of adoption factors. The next section reviews existing literature on IoT and ML applications in healthcare, followed by presenting the research model. Sections 4 to 6 present the study methodology, results, and discussion, respectively. The final sections of the paper acknowledge its limitations, discuss its implications, and conclude the paper, providing recommendations for further research and practice.

## 2 Literature review

### 2.1 IoT in healthcare

The IoT has transformed the healthcare industry by introducing innovative monitoring, diagnosis, and treatment solutions, ranging from chronic disease management to routine symptom monitoring (e.g., for pulse and hypertension). In the context of diabetes care, IoT tools can monitor blood glucose much more effectively and accurately than legacy solutions, which also do not share data live with integrated systems for monitoring and coordinating efforts (6), largely due to Wi-Fi connections across clinical, home, and even remote (i.e., smartphone-enabled) settings (7). While such solutions can be extremely useful when deployed effectively, it must be noted that IoT systems frequently suffer from malfunctions and drawbacks, and they may generate inconsistent or inaccurate data about patients (8). Consequently, more research is needed to develop healthcare integration of such tools to achieve objectives on the ground, as well as to improve related technologies in themselves, to enable great strides in chronic disease management, including ped-D.

### 2.2 Monitoring and analytics for responsive and predictive care

The whole IoT paradigm is fundamentally based on data from sensors being communicated instantly, including in managing diabetes, such as regular measurements of blood glucose, physical activity, and pulse data etc. Modern digital solutions can collect extensive, accurate data in real time (e.g., fit bands measuring the pulse and physical activity levels), offering vast quantities of high-quality data to HCPs that would be unimaginable a few years ago. Detecting problematic issues and emergencies can accelerate the initiation of appropriate responses, maximising health outcomes and health system efficiency. As noted by (9), smart monitoring using neural networks can offer analytics in addition to gathering data *per se*, enabling the prediction and categorisation of risks.

In addition to improving care for individuals, including tailored recommendations, IoT-integrated ML algorithms can help anticipate population-level trends in health needs, helping plan services more effectively, giving a fillip to preventive care. As a result of such advantages, IoT technologies are essential to improve diabetes care and management (10).

### 2.3 Non-invasive tools and wearable technologies

Wearables are increasingly popular due to their non-invasive (and largely consumer-driven) nature, and their impressive capabilities to monitor fundamental parameters of patients' physiological condition. Constant monitoring of the pulse, sleep, and activity tracking are relatively inexpensive staples of commercial fitness bands, while innovative add-ons, including glucose monitoring technology and insulin implants, can greatly improve diabetes management in patients' daily lives (11). Optical and electrochemical sensors in emerging wearables are far superior to former staples of diabetes care (12). For example, enzymic blood glucose monitoring was burdensome and progressively less accurate for patient care, but nowadays, “near-infrared” monitoring tools for blood glucose and insulin implants are far more convenient and clinically safe for patients, improving their health, safety, satisfaction, and personal lifestyle freedom (13).

### 2.4 System usability and user engagement

Technological “usability” is an essential feature of any device or system, including health-related IoT systems, which ought to be attractive and easy for target users to employ (e.g., patients, their family caregivers, and HCPs). It is particularly important that extensive technological learning, training, and knowledge are not required for patients, especially paediatric ones (where relevant), and that tools be as simple and easy to use as possible, to achieve the desired clinical outcomes. For instance, simple visualisations of blood glucose levels can intuitively help patients plan their children's nutrition (14). This pertains to the issue of user engagement with the technology, without which even the best IoT system would be useless. Continuous and real-time monitoring and timely alerts increase engagement, which is very important with ped-D (15).

### 2.5 Data processing and energy requirements

Collecting, communicating, and analysing large volumes of data (e.g., from sensors) in the IoT entails a very high energy demand, which can be ameliorated by the use of “fog computing”, whereby local (“edge”) devices and networks can undertake large proportions of necessary computing to reduce overhead on central systems, and rendering them more agile and responsive (42). In this regard, the wearables used by patients, and devices in patients' homes etc., should be designed for maximum energy efficiency, with minimal downtime and recharging, in order to be as safe and clinically effective as possible. This can also reduce ancillary financial costs incurred by people with diabetes and their families (i.e., minimising their power bills) (15). IoT health system can be optimised by processing data locally (at the “edge” of use), and by targeted extraction of required features, which reduce energy costs, and increase sustainability.

### 2.6 IoT adoption barriers

Theoretically, the obvious advantages of healthcare IoT, including those adumbrated above, are self-evident. However, numerous practical barriers can hinder their adoption. The most fundamental barriers are prosaic issues of cost and connectivity. Unfortunately, resource-constrained and geographically remote areas and service users may lack the commensurate financial resources and technological infrastructure to support such applications, despite them otherwise being poised to attain the greatest advantages from such solutions. Aside from such fundamentals, IoT systems themselves can have problems in the consistency and accuracy of data they gather and produce, which could undermine the quality and safety of care (e.g., a malfunctioning sensor for a critical clinical symptom) (16).

In diabetes management, the most obvious issue in this regard is glucose monitoring, for which precision is the most overwhelmingly important aspect of management, which also entails appropriate education and training for service users in addition to traditional “diabetes” education (i.e., service users must be trained in how to use and maintain health monitoring devices in their homes and daily lives). Relatedly, the most fundamental requirement of any system is that it works, yet IoT systems often fail to deliver reliable and consistent performance, which reduces their effectiveness (8).

Consequently, IoT hardware, software, and networks need to be continually improved to the maximum extent possible (17). This also applies to data privacy and security protections. Health systems are notoriously vulnerable to cyber threats, ranging from unauthorised data sharing to cyber-attacks, and patients' sensitive health data (among other types of data) in health communications must be inviolable at all times, with only approved and appropriate access to authorised parties. Advanced encryption and data storage techniques may ameliorate such concerns, albeit they remain serious problems for the whole IoT landscape (14).

### 2.7 Mixed method studies in healthcare technology research

Technology adoption in healthcare typically adopts one of the various well-known theoretical adoption models, but relating this to particular healthcare needs, to generate outcomes amenable to evidence-based practice, means that using mixed methods can be particularly advantageous. In this study, combining qualitative insights and quantitative data facilitates the evaluation of underlying adoption theories in relation to real IoT applications for ped-D management. There is extensive literature attesting to the value of mixed methods when exploring wearables in chronic disease management. For instance, Schretzlmaier et al. (18) explored mHealth adoption in chronic disease management utilising a quantitative cross-sectional survey and qualitative interviews with patients and HCPs. Timpel et al. (19) used iterative mixed methods, with an umbrella review, qualitative analysis of content, and a quantitative survey to analyse telehealth services for hypertension and diabetes management. Qualitative interviews and quantitative surveys of the wearers of wearables have been used in many studies (20, 21).

## 3 Research model

### 3.1 Overview

**Figure 1:**
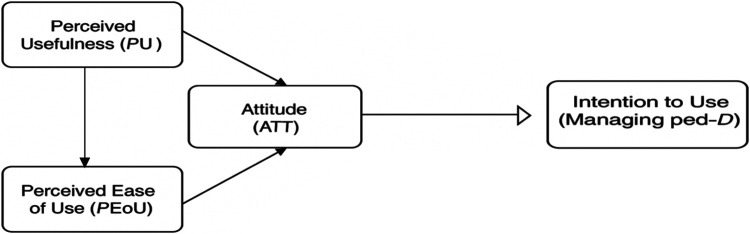
Conceptual model factors in smart healthcare for ped-D.

This study adopted the Technology Acceptance Model (TAM) (22) to study IoT use in diabetes management, pertaining to smartphone applications. This study's conceptual model (as displayed in Figure 1) is based on the TAM factors of Perceived Usefulness (PU) and Perceived Ease of Use (PEoU), “Attitude” (ATT) as a mediating variable, and managing ped-D (i.e., intention to use) as the dependent variable. Figure 1 shows the relation between variables, followed by a more summary explanation of the variables. All latent variables in this model are specified as reflective, consistent with the TAM framework.

### 3.2 Perceived usefulness (PU)

PU pertains to the subjective opinion formed by users of a technology that it is useful for their purposes. It is not necessarily the same as “usefulness” *per se*, and although user experiences of actual technology use tend to be decisive in their ultimate evaluation, they may not use the technology in the first place if they do not perceive that it will be useful for them, including HCPs and patients. The utility for the latter is judged primarily by the system's contribution to monitoring clinically relevant factors of ped-D, such as blood glucose, hypertension, physical activity, and overweight (23). Nowadays, patients using advanced health technologies typically expect individualised, tailored solutions and advice from systems, especially identification of risks (24).

PU can be increased by the capability of the IoT Management System (IMS) to produce reports for both HCPs and patients themselves about clinical data, including general health history, in addition to sensor-gathered incidental data on patients' prognosis, and socio-demographic factors. Consequently, tailored recommendations can be made, such as appropriate behavioural modifications or screening for preventive care (e.g., a sudden or indeed gradual reduction in a patient's physical activity levels may signal the need for a diabetic foot exam). Consequently, IoT can increase de facto communication among service users and HCPs, helping improve stakeholder contributions to decision-making, especially to kickstart preventive care (25). Consequently, the following hypothesis is tested:

**H1:** Perceived usefulness (PU) positively influences attitudes towards the use of IoT Management System (IMS) for Paediatric Diabetes (ped-D).

### 3.3 Perceived ease of use (PEoU)

While PU refers to users' perception of the utility inherent in a technology, PEoU pertains to how easy (or difficult) they think it will be for them to use in practice (5). With regard to the IMS, if users perceive that it is simple and straightforward to use (albeit with some basic instruction, e.g., from HCPs), this can facilitate its adoption and actual use. Conversely, if they perceive that it is difficult or cumbersome to use, they will shun it (26). Physical features and comfortableness (e.g., a tactile and aesthetically appealing interface) are instrumental in this, in addition to the functional attributes of the technology *per se* (27). Considering this dimension, we tested the following hypothesis:

**H2:** Perceived ease of use (PeoU) positively influences attitudes towards the use of IoT Management System (IMS) for Paediatric Diabetes (ped-D).

There is also an additional pathway reflects the well-documented TAM finding that users who perceive a technology as highly useful also tend to perceive it as easier to use, as their motivation to engage with it reduces perceived effort (5, 28). Accordingly the following hypothesis is tested:

**H3**: Perceived usefulness (PU) positively influences Perceived ease of use (PeoU) of the use of IoT Management System (IMS) for Paediatric Diabetes (ped-D).

### 3.4 Attitudes (ATT) and managing paediatric diabetes (ped-D)

Building on PU and PEoU, ATT signifies users' general receptivity or antipathy to the IoT technology, represented in ideation, opinions, and convictions concerning potential or actual use (29). People's ATT can be anywhere on a continuum from being open and proactively willing to try and use technology on an ongoing basis, through ambivalence and apprehension requiring encouragement, to outright hostility and rejection (and individuals can reflect differing ATT at different times). The more people engage with the technology, the more willing their ATT will be (presuming it meets their practical needs, and satisfies PU and PEoU), and greater familiarity and skill in use cascade into sustained use in practice (30).

As presented in Figure 1, the dependent variable is the intention to use the IMS in ped-D management, which is hypothesised to be affected by the variables PU, PEoU, and ATT. The adherence of patients (and their parents) depends on their intention to use the intervention, which can improve clinical protocol adherence and clinical outcomes. Health technology adoption can be particularly facilitated by PU and PEoU in relation to data and personalisation features (24, 25). In addition to features of technologies themselves, such technology adoption factors are also determined by users' personal attributes, which in the context of ped-D management include the openness of patients and their families to new things (31, 32), and age (28, 33). ATT toward IoT technologies in general mediates the way in which these diverse instrumental factors enable the IMS to contribute to ped-D management (29, 30). Based on this we test the following:

**H4:** Attitudes (ATT) towards the use of IoT Management System (IMS) positively influences the effectiveness and adoption of this system for managing Paediatric Diabetes (ped-D).

## 4 Materials and methods

Quantitative survey and qualitative interview methods were used in this mixed methods study, triangulating generalisable and in-depth date pertaining to the IMS for managing ped-D. The quantitative data from survey responses was analysed using Fuzzy-Set Qualitative Comparative Analysis (fsQCA) and Partial Least Squares Structural Equation Modelling (PLS-SEM). PLS-SEM was selected as a non-parametric method that reliably performs with smaller sample sizes and does not assume multivariate normality (34). The minimum sample size required for PLS-SEM analysis is determined by the 10-times rule, which requires at least ten observations per each construct (34).This approach is common in research on health information systems, with a view to discerning existential phenomena and contextual relationships between studied variables (35, 36).

The self-administered quantitative paper survey was distributed to 90 parents of ped-D patients (with either type of diabetes). It had two sections, the first of which introduced the participants to the goals of the project, covering wearables and health technology, leading into the IMS for managing ped-D. Accordingly, following this introduction general knowledge of IoT-related tools was assumed rather assessed as a formal eligibility criterion. The second section comprised 20 items in total, ascertained participants' personal readiness for the IMS (i.e., related to ATT, PU, and PEoU). Items were answerable using a Likert-type scale, with five options (from 1 “strongly disagree” to 5 “strongly agree”). The full list of survey items is provided in Supplementary Data Sheet 1. This study employs a cross-sectional design, whereby all data were collected at a single point in time. Accordingly, the structural relationships identified using both PLS-SEM and fsQCA should be interpreted as exploratory rather than strictly causal (34).

Semi-structured qualitative interviewees comprised 10 HCPs, including diabetes educators, endocrinologists, and paediatricians, exploring their perceptions of managing ped-D using the IMS. Questions reflected the studied constructs pertaining to their intention to use the system, including PU, PEoU, and ATT. The full interview guide, including all predefined questions, is provided in Supplementary Data Sheet 2.‏ Interviews were conducted in person by the research team and lasted approximately 20–30 min each.

Both techniques were used to test the study hypotheses presented above. Quantitative data analysis was conducted using SmartPLS 4 software to ascertain the structural relationships among the variables studied (34). fsQCA was used for the ascertainment of causality relating to adoption intention, using Python in alignment with fsQCA 3.0 software (37). Qualitative data analysis of interviews used manual thematic analysis, whereby audio recordings were transcribed and were repeatedly read to achieve data immersion, then codes were assigned to recurrent concepts, which were later grouped into themes reflecting the TAM constructs (38). Thematic saturation was monitored throughout data collection and considered achieved when no new themes emerged from successive interviews (39).

### 4.1 Sample description

Purposive sampling was used to recruit those caring for and managing ped-D, resulting in the recruitment of 90 parents of children and adolescents with both types of DM while no age criterion was formally applied, and 10 HCPs, including diabetes educators, endocrinologists, and paediatricians. Participant recruitment was undertaken at 3 diabetic and paediatric clinics in Amman, the capital city of Jordan, where over 50% of the national population resides. Purposive selection of parents was applied, whereby participants had high engagement with the diabetes management of their child (self-identifying as the main family caregiver) and basic familiarity with IoT tools, established through the introductory section of the survey. HCPs were selected according to their professional specialisation and direct experience of ped-D care. Recruitment was conducted by the research team, with the assistance of clinics staff who helped identify eligible participants during routine appointments. The main demographic and professional profiles of the participants are outlined in Tables 1, 2. Ethical approval for this study was granted by the Institutional Review Board (IRB) at the University of Jordan (Decision No. 567/2025), in accordance with the Declaration of Helsinki and the International Council for Harmonization (ICH) guidelines. Formal written informed consent was obtained from all participants before commencing data collection.

**Table 1:** Demographic characteristics of parents.

Variable	Category	Frequency (*N*)	Percentage (%)
Age (years)	28–34	28	31.1
	35–39	19	21.1
	40–44	13	14.4
	45–50	30	33.3

**Table 2:** Professional characteristics of HCPs.

Variable	Category	Frequency (*N*)	Percentage (%)
Specialty	Diabetes Educator	3	30.0
	Paediatrician	2	20.0
	Endocrinologist	3	30.0
	Other	2	20.0
Years of experience	<10	2	20.0
	10–19	3	30.0
	20–29	3	30.0
	30+	2	20.0

## 5 Results

### 5.1 PLS-SEM analysis

SmartPLS 4 software was used to analyse the hypothesised structural relationships among the TAM constructs in the PLS-SEM analysis. Following the standard two-step approach, the process began with evaluating the measurement model, followed by the evaluation of the structural model, whose measurement reliability convergent validity was determined according to Average Variance Extracted (AVE), Composite Reliability (CR), and Cronbach's *α* coefficient. Discriminant validity was determined as per the Fornell-Larcker criterion. Path coefficients of the structural model were evaluated, with significance determined through 5,000 bootstrapping procedures and the calculation of *R*² values (for power of explanation) (34).

#### 5.1.1 Measurement model

Analysing the measurement model pertains to exploring the hypothesised relations between the constructs (ATT, PeoU, PU, and Intention to Adopt/Use the IMS (IAU). Table 3 shows the AVE, CR, Cronbach's α, and standardized factor loadings for all constructs, indicating good reliability and internal consistency, as per the CR data (0.83–0.87). All constructs' AVE exceeds 0.60, supporting convergent validity. The measurement model's reliability is supported by Cronbach's *α* coefficients, which range from 0.79 to 0.84, indicating adequate internal consistency and reliability for all constructs.

**Table 3:** Construct validity—standardized factor loadings, Cronbach's α, CR, and AVE values.

Construct	Standardized Factor Loadings	Cronbach's α	CR	AVE
PU	PU1 = 0.8, PU2 = 0.7, PU3 = 0.75, PU4 = 0.82, PU5 = 0.78	0.84	0.89	0.68
PEoU	PEoU1 = 0.78, PEoU2 = 0.72, PEoU3 = 0.79, PEoU4 = 0.85, PEoU5 = 0.76	0.81	0.87	0.63
ATT	ATT1 = 0.82, ATT2 = 0.79, ATT3 = 0.75, ATT4 = 0.83, ATT5 = 0.77	0.85	0.90	0.72
IAU	IAU1 = 0.76, IAU2 = 0.74, IAU3 = 0.78, IAU4 = 0.81, IAU5 = 0.79	0.86	0.91	0.74

#### 5.1.2 Structural model

**Figure 2:**
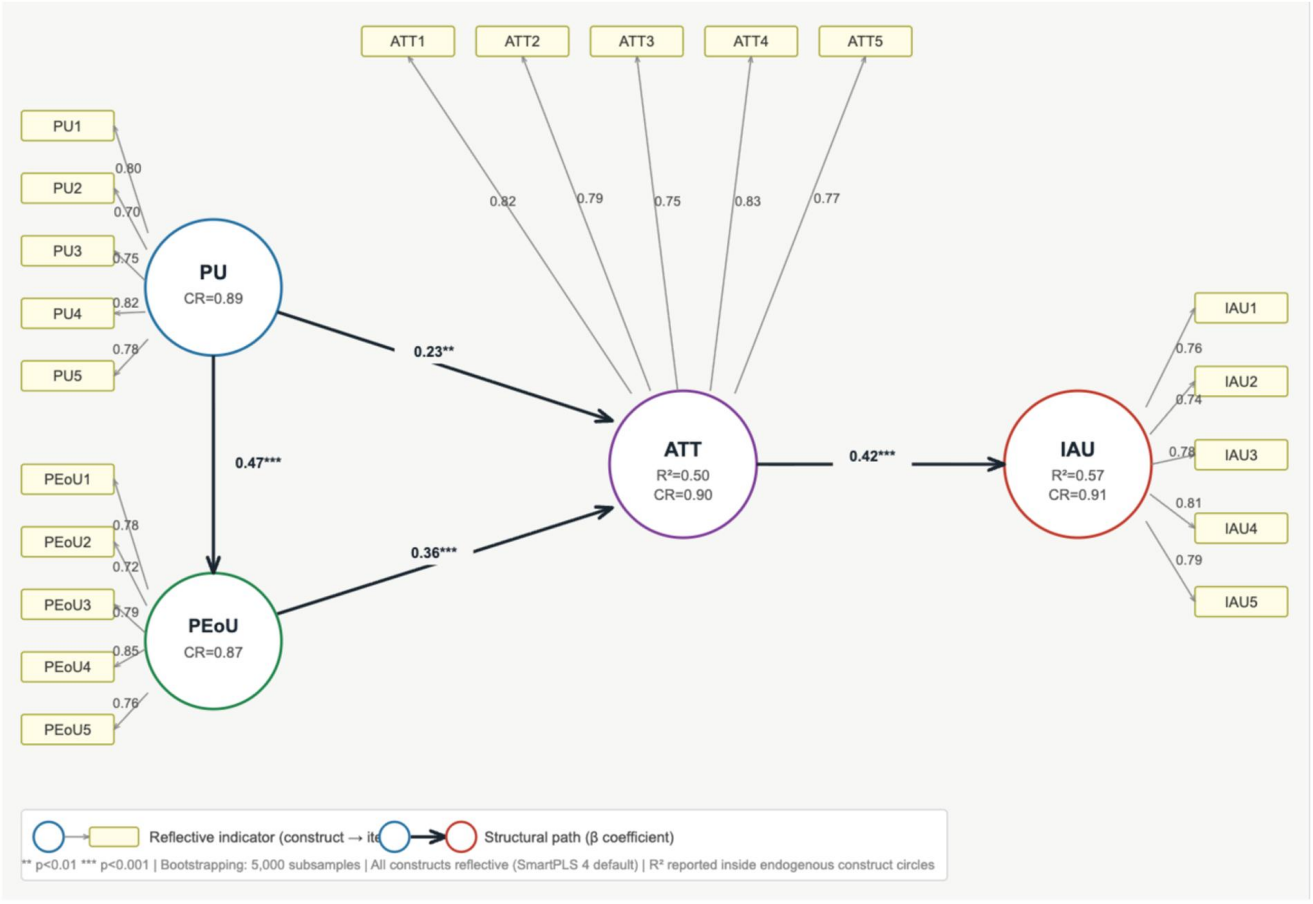
Model analysis.

The structural model investigates the relationships between the constructs identified in the measurement model. In this instance, it examines the relationship between adoption intentions and the use of IoT-based diabetes management systems for children, considering the influence of views on usefulness, convenience, and ATT. The structural model was tested using a bootstrapping procedure with 5,000 subsamples. Figure 2 illustrates the findings of the model analysis. The results of testing the hypothesised relationships between the studied constructs are summarised in Table 4, showing that all of the hypothesised relationships (i.e., H1, H2, and H3) are supported.

**Table 4:** Results of path analysis and hypotheses testing.

Hypotheses	Path Coefficients	T Statistics	*p* Values
PU ⇒ ATT	0.23	2.89	<0.01
PEoU ⇒ ATT	0.36	4.82	<0.001
PU ⇒ PEoU	0.47	5.62	<0.001
ATT ⇒ IAU	0.42	5.23	<0.001

The model yielded R² values of 0.50 for ATT and 0.57 for IAU, indicating substantial explanatory power. The Variance Inflation Factor (VIF) values are below 3, indicating no multicollinearity issues.

### 5.2 fsQCA

To enhance the variance-based findings of the SEM model, and further examine the causal complexity driving adoption behaviour, fsQCA was conducted, to uncover alternative causal paths sufficient for achieving a high IAU IoT-enabled solutions for managing ped-D.

#### 5.2.1 Calibration and truth table construction

All constructs were measured using five-point Likert scales and calibrated into fuzzy-set membership scores through the direct method. The calibration thresholds were set at 0.95 for full membership (Likert score of 5), 0.50 for the crossover point (score of 3), and 0.05 for full non-membership (score of 1). This transformation of raw values into fuzzy scores follows established guidelines for fsQCA in social and health sciences (36, 37).

A truth table was constructed by splitting the calibrated scores at a threshold of 0.50. Configurations with fewer than two cases were excluded, and a consistency threshold of 0.80 was applied to identify combinations of causal conditions that reliably lead to high adoption intention. The analysis focused on the three key antecedent conditions from the TAM model: PEoU, PU, and ATT.

#### 5.2.2 Configurational solutions

The analysis identified two causal configurations sufficient for achieving a high IAU IoT solution. These configurations are presented in Table 5, which includes consistency and coverage metrics.

**Table 5:** Configurations sufficient for high IAU IoT (TAM model).

Configuration	PEoU	PU	ATT	Coverage		Consistency
				Raw	Unique	
C1	●	●	●	0.68	0.59	1.00
C2	⊗	●	●	0.20	0.11	1.00

● = presence of condition; ⊗ = absence of condition.

The primary configuration (C1) indicates that the simultaneous presence of high PEoU, high PU, and positive ATT constitutes a sufficient condition for high IAU. C2 posts an alternative, whereby low PEoU can be offset by high PU and ATT, which are sufficient in themselves to engender IAU. It is important to highlight that in fsQCA, constructs are treated as conditions rather than endogenous variables in a causal chain as in the PLS-SEM. This distinction reflects the roles of the two analytical approaches: PLS-SEM examines the causal relationships, while fsQCA identifies combinations of conditions sufficient for the outcome.

#### 5.2.3 Predictive validity

The relationships between the variables were analysed to determine their generalisability and robustness by dividing the data into training and “holdout” samples (with 70% and 30%, respectively). Subsequently, the dominant C1 configuration was applied for the holdout sample, revealing complete consistency (100%) for matched cases (*n* = 25), affirming the model's stability for subsamples, and its predictive power.

### 5.3 Interviews

The qualitative interview results reflecting perceptions of ped-D HCPs and their ATT concerning wearables' adoption are presented in Table 6, organised by themes aligned with the TAM constructs examined in the quantitative analysis. Two main conclusions are evident, as adumbrated below, lending additional support to the statistical analyses affirming the research hypotheses; representative quotations are provided for each theme to substantiate the thematic findings:

**1. PU and PEoU:** Participants clearly perceived the intended advantages of using wearables for ped-D, as per the large mean scores for PU and PEoU, indicating a latent receptivity (i.e., positive ATT, as discussed below) to using the IMS to improve patient monitoring and quality of care. Doctor 1 stated: “These devices provide continuous glucose monitoring, which is crucial for managing a child's diabetes effectively”. Doctor 6 observed: “The user interface of these devices is intuitive and simple to follow”.
**2. ATT and IAU:** High mean scores for ATT and IAU underscore how positive predispositions to using the IMS intersect with plans to imminently integrate wearables into ped-D care. This also reflects a professional openness to innovative solutions in general among HCPs. Doctor 4 stated: “Ready and prepared. I believe it is important for both parents and healthcare providers to stay updated on technological advancements”. Doctor 8 noted: “They offer the flexibility to monitor at home, leading to fewer emergencies”.

**Table 6:** Summary of salient issues from HCP interviews.

Question themes	Most frequent theme	Frequency
1. Ease of use, User-friendly design, Simple integration, Minimal training required, Real-time tracking	PeoU (User-friendly design)	6
2. Continuous monitoring, Accuracy of data, 24/7 tracking, Improved glucose control, Stable glucose levels	PU (Continuous monitoring)	7
3. Readiness to adopt, Positive outlook on tech, Modern management, Support for advancements, Excitement for future tech	ATT (Readiness to adopt)	6
4. Improved management, Better glucose control, Less stress for families, More accurate data, Fewer emergencies	IAU (Improved management)	8

The emergent thematic outcomes from analysing the interview data reveal that HCPs for ped-D patients have a high level of receptivity and optimism toward the IMS, and are eager to adopt the use of wearables. While this in itself is a positive indicator, potential obstacles raised by participants must be borne in mind, such as requirements for prerequisite employee training, integrating the IMS and commensurate devices in existing practices, and acceptance among service users themselves (i.e., children and their parents). Accommodating such issues from the outset (i.e., from system design phase) can avoid potential obstacles later, and enable the most clinically and cost-effective integration of wearables in ped-D management, achieving the best options for HCPs and service users.

## 6 Discussion

Using mixed methods, including quantitative survey data analysed using fsQCA and PLS-SEM, and semi-structured interviews with HCPs, has revealed important insights on IMS adoption for ped-D care, based on triangulated data pertaining to the TAM view of technology acceptance. PLS-SEM affirmed the applicable nature of the TAM, whereby both PU and PEoU were found to exert statistically significant impacts on ATT, and thus IAU. However, PU was found to be the most instrumental of the studied variables, whereby both PU and PEoU were found to exert statistically significant impacts on ATT and thus IAU.

These findings can be attributed to the key role played by ped-D patients' parents in managing the condition (e.g., monitoring symptoms in the home), which is an area where the IMS specifically seeks to improve service quality (i.e., making tasks easier for parents and safer and more effective for patients). ATT in relation to user perceptions is fundamentally affected by usability issues, including reducing anxiety, uncertainty, and the cognitive load associated with system use (24, 26). Previous studies similarly supported the utility of TAM to conceptualise healthcare implementations of technological solutions, including IoT applications (4, 5).

The fsQCA evaluation illuminated causative relations instrumental in IAU. The most intuitive path identified by path analysis underscored how positive PU, PEoU, and ATT were collectively conductive to optimal IAU, but that the latter could be driven even by positive ATT and PU alone (i.e., without necessarily having PEoU). This pertains to “equifinality”, whereby differing configurations of instrumental factors can attain the same end result (36, 37). Related to this asymmetry in causation, the fsQCA results suggest that, while highly instrumental, PEoU is not absolutely necessary, which can be attributed to intended users of the IMS being willing to accept certain limitations in *de facto* system features and use as long as they perceive that the attained benefits outweigh such limitations, which, in the studied context of ped-D, include reduced clinical visits. Such outcomes are in accordance with other healthcare technology applications in relation to adoption predispositions being affected by “compensatory behaviours” (35, 40).

The statistical analysis of questionnaire results were complemented by the thematic analysis of the semi-structured interviews with HCPs, who indicated the great potential for IMS to improve ped-D management, especially in relation to making it faster and easier to monitory blood glucose, issue customised alerts, and improve parental decisions. Relating to such factors, their narratives emphasised the importance of PU. The HCPs also suggested that identifying clinical symptoms and initiating early, tailored solutions could improve patients' prognosis and professionals' own confidence and performance, supporting the findings on PU's instrumentality from the SEM and fsQCA analyses. While usability concerns were acknowledged, they were typically considered to be manageable. HCPs indicated that practical training, technical support, and caregiver motivation can offset less-than-ideal interface design. This aligns with the fsQCA insight that PEoU is not always essential for adoption.

The mixed-methods design significantly enhanced the explanatory depth of this study by combining statistical validation, configurational analysis, and contextual insight. The PLS-SEM model validated the proposed linear relationships between key TAM constructs, showing that PU and PEoU significantly influenced ATTs and intentions to adopt IoT-based systems for managing ped-D. The outcomes provide a general overview of how the studied variables affect each other, with implications for diabetes care, while fsQCA specified conditional combinations conducive to adoption rates. The latter indicates that PEoU definitively supports adoption, but PU and ATT can achieve it in the absence of PeoU, and this equifinality suggests the need for flexibility and contextual adaptions relative to user behaviours.

The qualitative findings from the thematic analysis of interviews offered ancillary insights supporting the quantitative findings, triangulating them with the real clinical experiences of HCPs. Professionals emphasised the particular importance of system advantages including monitoring patient data and consequently reducing the need for clinical visits, and they underscored the essential impact of PU. They noted that barriers to system use could be ameliorated with commensurate training and IT support for system users, which relates to the fsQCA finding that PEoU is not absolutely necessary for positive IAU. The triangulation of methods increases the main outcomes' credibility, reflecting the multifaceted phenomena of healthcare technology adoption, relating to technology itself, user perceptions of it, and practical contextual settings, which collectively interact via sophisticated paths.

This study thus adds to the increasing body of research on digital health technological adoption, and demonstrates the utility of the TAM, which it extends upon with regard to analysis of complex variables' analysis with regard to HCPs' expert knowledge. It also indicates the value of the integration of multiple forms of analysis to study complex healthcare settings, to comprehend personnel and technological factors that collectively influence technology adoption behavioural responses.

## 7 Conclusions

### 7.1 Main outcomes

This mixed methods study offered a robust analysis of factors pertaining to the adoption of IoT-based systems for the management of ped-D, using fsQCA and PLS-SEM to analyse quantitative survey data, and thematic analysis of semi-structured interviews. The study demonstrated the ongoing relevance of the TAM while extending it through statistical analysis to identify configurational pathways among PU, PEoU, ATT, and IAU and their importance. PU and ATT were universally impactful, but PEoU was not absolutely essential (albeit it was instrumental in IAU). Furthermore, the fsQCA analysis illuminated how different configurations fostered IAU, which indicated that strategies for adoption and use ought to retain flexibility. Qualitative findings situated the findings in relation to clinical contexts, underscoring the importance of user trust and preparedness, while noting barriers to use and integration.

Collectively, the outcomes improve comprehension of IMS adoption and IoT integration for the management of chronic disease, and signal the need for sophisticated analyses of healthcare technologies. It is recommended for future research to study longitudinal implications and impacts, the perspectives of a broader user base, and the integration of systems with predictive analytics in order to assay the scalability and sustainability of such innovative systems in healthcare contexts.

### 7.2 Implications

The outcomes of this research on technological readiness for IMS to manage ped-D have important practical and theoretical implications. In terms of theory, our comprehension of healthcare technology adoption can be improved by using configurational analyses with structural equation modelling, complemented by qualitative insights. Analysis using fsQCA affirms the explanatory power of the TAM, identifying multiple adoption paths, supporting contextually sensitive user behaviour studies. This opens new directions for developing theory concerning configuration, alongside the classic affirmation of key predictors' applicability.

The essential instrumentality of PU and user ATT in driving adoption is affirmed, and users are prepared to use the IMS even if it has poor usability or high complexity *per se*, as long as it is effective in improving patient outcomes. Developers and HCPs should note the importance of clinical efficacy and not presume that consumer-oriented improvements (e.g., aesthetics) are paramount for service users. However, technical assistance to facilitate use is important, and necessary education and training on system use must be provided, including for HCPs as well as family caregivers and patients themselves. Furthermore, the IMS should be integrated with the applicable Electronic Health Record (EHR) system, with commensurate safeguards for user privacy and security. System design must accommodate these considerations to help promote adoption in clinical practice. Technologically, IoT and ML integration with predictive analytics can improve clinical efficiency by tailoring care and targeting early interventions where necessary, optimising resource utilisation by health systems to manage chronic conditions such as diabetes with the maximum efficiency.

### 7.3 Limitations

The main limitation of this research is its small sample size, for both the quantitative survey and the qualitative interviews; the relatively small number of survey participants and interviewees undermines the wider applicability of the results, as does the restriction to the geographical context of Amman, Jordan. Additionally, the incomplete sociodemographic characterisation of the study sample is limited, as paediatric patients age was not recorded and their parents sociodemographic such as gender, employment and digital literacy. Furthermore, focusing on ped-D does not account for potential IMS use for the management of other chronic conditions, whose dynamics pertaining to technology adoption can vary due to clinical symptoms, monitoring, and user engagement. Additionally, we did not thoroughly analyse the technological availability of service, and infrastructural barriers such as lacking continuous, high-quality internet access which would be fundamental to adoption, particularly for the use of wearables in home settings, especially in low-income and geographically remote contexts. Finally, albeit we used a robust and normative mixed-methods approach, the cross-sectional design of this study is considered a fundamental methodological concern. The absence of longitudinal data precludes the ability to detect long-term changes in perceptions and use associated with IMS effectiveness.

## Data Availability

The raw data supporting the conclusions of this article will be made available by the authors, without undue reservation.

## Supplementary material

Supplementary Data Sheet 1 Full survey instrument items for the quantitative questionnaire, organised by TAM construct (PU, PEoU, ATT, IAU), including item wording, standardised outer loadings, and source references.

Supplementary Data Sheet 2 Semi-structured interview guide used with healthcare professional (HCP) participants, including all five predefined interview questions aligned with the TAM constructs.
