# Supplementary material for: IoT-enabled solutions for paediatric diabetes: examining consumer readiness and adoption factors
Source: Front Digit Health. 2026 Jul 8;8:1747032. doi: 10.3389/fdgth.2026.1747032 (PMC13388807; doi:10.3389/fdgth.2026.1747032)
Supplement: Supplementary Data Sheet 1 — Full survey instrument items for the quantitative questionnaire, organised by TAM construct (PU, PEoU, ATT, IAU), including item wording, standardised outer loadings, and source references. [file Datasheet1.docx]

**Appendix A**

***Survey Instruments***

Table A1 presents the full list of items used in the second section of the questionnaire, organised by latent construct. All items were measured using a five-point Likert scale (1 = Strongly Disagree; 5 = Strongly Agree).

**Table A1**

*Survey Items*

| **Code** | **Item** | **Source** |
| --- | --- | --- |
| **Perceived Usefulness (PU**) | | |
| **PU1** | Using the IoT system would improve my ability to monitor my child's blood glucose. | Davis (1989); Al-rawashdeh et al. (2022) |
| **PU2** | Using the IoT system would enhance the effectiveness of managing my child's diabetes. | Alhasan et al. (2022) |
| **PU3** | The IoT system would provide timely alerts that help me respond quickly to changes in my child's condition. | Hossain et al. (2021) |
| **PU4** | The IoT system would give me greater control over my child's health management. | Al-rawashdeh et al. (2022) |
| **PU5** | Overall, I find the IoT system useful for managing my child's diabetes. | Davis (1989); Arfi et al. (2021) |
| **Perceived Ease of Use (PEoU)** | | |
| **PEoU1** | Learning to use the IoT system for my child's diabetes care would be easy for me. | Davis (1989) |
| **PEoU2** | I find the IoT system's interface clear and understandable. | Davis (1989); Alharbi et al. (2020) |
| **PEoU3** | Interacting with the IoT system does not require a lot of mental effort. | Davis (1989) |
| **PEoU4** | I would be able to use the IoT system without needing extensive technical support. | Nuryakin et al. (2023) |
| **PEoU5** | Overall, I find the IoT system easy to use. | Davis (1989); Bestepe & Yildirim (2022) |
| **Attitude (ATT)** | | |
| **ATT1** | Using the IoT system for my child's diabetes management is a good idea. | Shuhaiber et al. (2019) |
| **ATT2** | I feel positively about using the IoT system in my child's daily care routine. | van Deursen et al. (2021) |
| **ATT3** | I believe the IoT system would make diabetes management more manageable for our family. | Bhadauria & Chennamaneni (2022) |
| **ATT4** | I am enthusiastic about the prospect of using IoT technology in my child's healthcare. | Gokcearstan et al. (2024) |
| **ATT5** | My overall attitude toward the IoT system for paediatric diabetes management is favourable. | Shuhaiber et al. (2019) |
| **Intention to Adopt/Use (IAU)** | | |
| **IAU1** | I intend to use the IoT system to monitor my child's diabetes in the near future. | Hossain et al. (2021); Alhasan et al. (2022) |
| **IAU2** | I plan to adopt the IoT system as part of my child's regular diabetes care. | Arfi et al. (2021); Duarte & Pinho (2019) |
| **IAU3** | I would recommend the IoT system to other parents managing their child's diabetes. | Almansour et al. (2023) |
| **IAU4** | Given the opportunity, I would integrate the IoT system into my child's current diabetes care routine. | Arfi et al. (2021) |
| **IAU5** | I am willing to commit to using the IoT system on an ongoing basis for my child's health monitoring. | Duarte & Pinho (2019) |

Note. All constructs are specified as reflective, consistent with TAM theoretical assumptions (Hair et al., 2019).
