# Supplementary material for: IoT-enabled solutions for paediatric diabetes: examining consumer readiness and adoption factors
Source: Front Digit Health. 2026 Jul 8;8:1747032. doi: 10.3389/fdgth.2026.1747032 (PMC13388807; doi:10.3389/fdgth.2026.1747032)
Supplement: Supplementary Data Sheet 2 — Semi-structured interview guide used with healthcare professional (HCP) participants, including all five predefined interview questions aligned with the TAM constructs. [file Datasheet2.docx]

**Appendix B**

**Interview Questions**

The following five predefined questions were used to guide each interview.

**1. To what extent do you agree that wearable technological devices, such as smart patches placed on the hands of children with diabetes, are easy to use?**

**2. To what extent do you agree that wearable technological devices (such as smartwatches, smart bracelets, smart patches for monitoring blood sugar levels, and others) for children with diabetes are useful in managing their condition?**

**3. To what extent do you agree that you consider yourself ready to experience using modern technologies (such as wearable devices) in managing and monitoring your child's diabetes?**

**4. Do you agree that wearable technological devices have helped you improve the management and monitoring of your Patient’s diabetes?**

**5. To what extent do you agree that the age of the parents may influence their attitudes towards using modern technologies, such as wearable devices, in managing their children's diabetes?**
